# Supplementary material for: Spatial variations and predictors of overweight/obesity among under-five children in Ethiopia: A geographically weighted regression analysis of the 2019 Ethiopian Mini Demographic and Health Survey
Source: PLoS One. 2024 Oct 14;19(10):e0312025. doi: 10.1371/journal.pone.0312025 (PMC11472954; doi:10.1371/journal.pone.0312025)
Supplement: Attachment — Submitted filename: Overweight review.docx [file pone.0312025.s002.docx]

Overweight review

**About the novelty of the title**

I can see that the title ha has been published through various ways. Hence, the authors should have a justification regarding it (few of them are listed below based on EDHS 2019,)

1. Spatial variation and determinants of underweight among children under 5 y of age in Ethiopia: A multilevel and spatial analysis based on data from the 2019 Ethiopian Demographic and Health Survey
2. Spatial distribution and associated factors of severe malnutrition among under-five children in Ethiopia: further analysis of 2019 mini-EDHS
3. Prevalence of overweight/obesity and associated factors among under-five children in Ethiopia: A multilevel analysis of nationally representative sample

**Title**

**Suggestions for Improvement:**

Consider including the specific timeframe of the study. For example, if the data collection period is mentioned, it can provide additional context and make the title more precise.

To enhance clarity, consider specifying the exact type of geographically weighted regression analysis utilized in the study. This will provide more detail on the specific approach used and could be of interest to readers familiar with different variations of geographically weighted regression.

Revised Example:

"Spatial variations and associated factors of overweight/obesity among under-five children in Ethiopia: a geographically weighted regression analysis of 2019 DHS data"

**Abbreviation/acronyms utilization**

Starting from your abstract the authors should re-arrange the utilization of abbreviations and acronyms accordingly

**Introduction**

The introduction section provides an overview of the global prevalence of childhood obesity/overweight and highlights the increasing burden in low-income countries, specifically focusing on Ethiopia. Here are some comments on the introduction:

Outdated references: The introduction section relies on references that are outdated, as indicated by the reference numbers (e.g., reference numbers 1, 2, 3, etc.). It is important to ensure that the references used are current and reflect the latest available data and research on the topic. Some of the reference also incorrectly cited. The authors should find the primary source and let the authors acknowledge them

**Lack of global to local context**: The introduction does not provide a clear transition from the global context of childhood obesity/overweight to the specific local context of Ethiopia. It would be beneficial to link the global prevalence statistics to the situation in Ethiopia and highlight the unique factors contributing to childhood obesity/overweight in the country.

**Limited attention to childhood obesity in Ethiopia**: The introduction suggests that Ethiopia does not consider childhood obesity as an emergent public health concern and mentions limited attention given to this issue. However, they did not put the references for their investigation or justification, and it would be helpful to provide more context or evidence to support this claim and explain the potential reasons for the lack of attention.

**Need for spatial analysis:** The introduction mentions that previous research in Ethiopia did not consider the spatial distribution of overweight/obesity and spatial regression analysis. It correctly highlights the importance of spatial analysis for policymakers to allocate resources effectively. However, it would be beneficial to provide more context on why spatial analysis is specifically relevant in the Ethiopian context and how it can contribute to addressing the issue.

**Research gap:** The introduction identifies a research gap by stating that few studies have explored community-level factors influencing overweight/obesity in Ethiopia. This highlights the need for the current study and its contribution to filling this gap. However, it would be helpful to provide more context on the specific aspects of community-level factors that have been underexplored in previous research.

**Importance of the study**: The introduction briefly mentions that the study aims to determine the spatial variations of overweight/obesity and its associated factors among under-five children in Ethiopia. It also emphasizes the potential implications of the study for policymakers, health planners, researchers, and health professionals. However, it would be beneficial to provide more specific details on how the findings of this study can inform policy and practice in Ethiopia.

**Methods and materials**

**Study design, period, and area**

What is EMDHS

Please clearly define the study setting, and related concepts or feeding practices and sources in the country. It will help readers

Try to put regionals states based on their order, and indicate there also another additional emerging region

**Source and study population**

The authors declared that their population was from 0-59 months. The question is

Body Mass Index (BMI) is generally not measured for newborns. BMI is a measure of body fat based on height and weight, typically used in older children, adolescents, and adults. For newborns, different measures are used to assess growth and development, such as weight-for-age, length-for-age, and weight-for-length percentiles. These measures help evaluate whether a newborn's growth is within the expected range for their age and sex.

BMI calculations require both height and weight measurements, which are not practical or accurate to obtain in newborn infants. Additionally, BMI is not considered an appropriate indicator of body fat for infants due to their rapidly changing body composition and growth patterns.

What about those children whose day of birth is missing or unknown

How did you manage other missing values. The authors should put the detail regarding their management

**Data collection tool and procedures**

In this section the author mentioned about other than the given topic

No sample size determination and sampling methods section

The authors need to create new session about sample size determination and sampling methods

Reference 14, 19…were not the appropriate references for the Data collection tool and procedures and outcome variable. Kindly use the correct one

**Data management and statistical analysis**

What does it mean “Using the STATA drop command in conjunction with a logical or conditional expression, we remove missing values from our analysis”.

**Autocorrelation**

Why the authors applied “The outcome variable has a Bernoulli distribution”. Have you appreciated that the continuous nature was also used in your model? Why did not use the poison one

**Results**

Do not use the new sentence with “of” and the like

How many unweighted participants did you included

Have the authors found primary clusters only in the SaTscan analysis

“Spatial scan analysis” what do you mean by “Spatial scan analysis” did you mean “Spatial SaTScan analysis”

**Discussion**

The provided discussion contains valuable information about the geographical variations in overweight/obesity and related factors among Ethiopian children under the age of five. However, there are a few areas that could be improved:

**Consistency in referencing**: The discussion refers to various studies to compare the prevalence of overweight/obesity in Ethiopian children, but the references are not consistently cited using proper citation format. It would be helpful to ensure that the references are cited correctly and consistently throughout the discussion. There are also miss citations. The author should have the primary source for their citation

**Clarity in reporting findings**: While the discussion provides some numerical findings regarding the prevalence of overweight/obesity in different regions, it would be beneficial to present the findings in a more organized and structured manner. Consider using tables or concise bullet-point format to clearly present the prevalence rates and comparisons between regions and previous studies.

**Explanation of findings:** The discussion mentions various factors that may explain the observed variations in overweight/obesity, such as delivery via cesarean section, socioeconomic status, and urban residence. It would be helpful to provide more detailed explanations for each factor, including the underlying mechanisms and potential implications for interventions or preventive measures.

**Expansion of limitations and implications**: The discussion briefly mentions methodological differences, cultural feeding practices, and nutrition assessment methods as possible explanations for the observed discrepancies in overweight/obesity prevalence. It would be beneficial to expand on these limitations and discuss their implications for future research or interventions in more detail. Furthermore, it was not clear how and why authors excluded missing values

**Consistency in language and terminology:** Some sentences in the discussion use different terminology or language styles. It is important to maintain consistency in terminology and language throughout the discussion to ensure clarity and coherence

Write clearly the implication of your study findings to the policymakers
