# Supplementary material for: Spatial variations and predictors of overweight/obesity among under-five children in Ethiopia: A geographically weighted regression analysis of the 2019 Ethiopian Mini Demographic and Health Survey
Source: PLoS One. 2024 Oct 14;19(10):e0312025. doi: 10.1371/journal.pone.0312025 (PMC11472954; doi:10.1371/journal.pone.0312025)
Supplement: Attachment — Submitted filename: Comment to reviewer.docx [file pone.0312025.s003.docx]

**Comment to reviewer**

**Abstract:**

1. **Result : please support by statistics the following result “The spatial distribution of overweight/obesity among under-five children in Ethiopia was clustered”**
2. **Please support by statistics the following finding in the result “In the geographically weighted regression analysis, urban residence, cesarean section, rich households, and male children were statistically significant factors.**
3. **conclusion: please put yours recommendation based on the result , means against the identified factors**
4. **what is the contribution of 12 authors in secondary analysis**

**Background**

1. **please put citation for the following statement “The majority of overweight and obese children live in developing countries, where the pace of increase is more than 30% greater than in industrialized countries.**
2. **The following statement is not clear please rewrite “Globally, the prevalence of children under five who are obese was 7% in 2012, and by 2025, it was expected to drop to less than 11% (5)” can we say 7% to 11 % is drop???.**
3. **Please rephrase the following statements “From 5.4 million in 1990 to 10.3 million in 2014, the number of overweight and obese children in Africa has almost doubled (2, 6)”**
4. **Rephrase the following statement as “6.8% of children between the ages of 0 and 59 months are overweight or obese, according to 26 Demographic and Health Surveys that have been carried out in the SSA since 2010 (7) “.you can rephrase as : according to the Demographic and Health Surveys conducted in SSA, about 6.8% of children between the ages of 0 and 59 months were overweight or obese in 2010.**

**Generally, the introduction is not well written, incoherence, and many of the statement were written without citation, my recommendation for the authors, please remove unnecessary details and put your evidence or citation for some of statements that reported without citation.**

**Methods:**

1. **When do you score "1," else marked as "0" for the outcome variable “Overweight/obesity “**
2. **The following statement is not clear “STATA was used to tabulate the weighted proportions of outcome variables and prospective predictor variables, which were then exported to Excel and imported into ArcGIS 10.6 for additional analysis”, please remove**
3. How the authors handle the two levels hierarchy nature of MEDHS data (multi-stage stratified cluster). That the Level one units were individual children in households and level two units were enumeration areas (community level). That Level one children were nested in the households, then the households were nested at the next higher level of enumeration areas ( community level). With is reality, Is Geographic Weighted Regression model is the appropriate model to identify the individual level and community-level factors as you claim in the bottom of your introduction???. I firmly recommend the authors to run multilevel mixed effect logistic regression rather Geographic Weighted Regression model to identify the individual level and community-level factors as you claim to identify factors at respective level.
4. **How you weight the variables**

**Results**

5. **Please, specify the** individual level and community-level factors **separately in result**
6. **Discussion :generally the discussion need rewriting and grammar correction**
7. **Please write the clinical and public health importance of this study ,just at end of discussion**
