# Supplementary material for: Spatial variations and predictors of overweight/obesity among under-five children in Ethiopia: A geographically weighted regression analysis of the 2019 Ethiopian Mini Demographic and Health Survey
Source: PLoS One. 2024 Oct 14;19(10):e0312025. doi: 10.1371/journal.pone.0312025 (PMC11472954; doi:10.1371/journal.pone.0312025)
Supplement: Attachment — Submitted filename: point by point response.docx [file pone.0312025.s004.docx]

**Date:** 08/07/2024

## **Dear editorial board member (s) of** **PLOS ONE journal**

The authors have been recalled to revise the manuscript entitled “Spatial variations and associated factors of overweight/obesity among under-five children in Ethiopia: a geographically weighted regression analysis of 2019 DHS data” identified by unique manuscript number: PONE-D-23-34960, which was submitted for publication. So, we received the editor(s)’ and reviewer's revision comments for the improvement of the manuscript before its publication.

Thank you for receiving comments, suggestions, directions, and questions from the editor and reviewer. As we said usually, we are very happy in receiving constructive and valuable comments for making a better manuscript. Accordingly, we have considered all the comments, questions, directions, and suggestions and provided a point-by-point response letter.

Finally, we have submitted all the required documents in their revised form. We hope that we have addressed all the suggestions, directions, and raised questions and if you believe that point(s) is not addressed, please let us know.

**Thank you very much to all editor (s), and reviewers**

**On the behalf of the authors**

**Yours sincerely,**

**Correspondence author**

**PLOS ONE academic editor**

**Point-by-point response letter**

**Reviewer comments 1**

**About the novelty of the title**

I can see that the title ha has been published through various ways. Hence, the authors should have a justification regarding it (few of them are listed below based on EDHS 2019,)

1. Spatial variation and determinants of underweight among children under 5 y of age in Ethiopia: A multilevel and spatial analysis based on data from the 2019 Ethiopian Demographic and Health Survey
2. Spatial distribution and associated factors of severe malnutrition among under-five children in Ethiopia: further analysis of 2019 mini-EDHS
3. Prevalence of overweight/obesity and associated factors among under-five children in Ethiopia: A multilevel analysis of nationally representative sample

**Author reply:** Thank you for your essential comment. Even though the titles was done by multilevel analysis, this study focused on Geographically Weighted Regression (GWR) analysis that allows for a nuanced understanding of the spatial variations in factors contributing to overweight among children under five, revealing how relationships between overweight prevalence and predictors like socioeconomic status, dietary patterns, physical activity, and environmental factors differ across geographic locations. This localized insight identifies specific hotspots and cold spots, enabling policymakers to tailor interventions to regional needs, thus optimizing resource allocation and intervention strategies. By providing detailed spatial patterns and enhancing understanding of underlying mechanisms, GWR supports the development of targeted, effective, and contextually appropriate health policies and programs.

**Title**

**Suggestions for Improvement:**

Consider including the specific timeframe of the study. For example, if the data collection period is mentioned, it can provide additional context and make the title more precise.

To enhance clarity, consider specifying the exact type of geographically weighted regression analysis utilized in the study. This will provide more detail on the specific approach used and could be of interest to readers familiar with different variations of geographically weighted regression.

Revised Example:

"Spatial variations and associated factors of overweight/obesity among under-five children in Ethiopia: a geographically weighted regression analysis of 2019 DHS data"

**Author reply:** thank you for your fruitful comment and based on your comment we revised the title accordingly.

**Abbreviation/acronyms utilization**

Starting from your abstract the authors should re-arrange the utilization of abbreviations and acronyms accordingly

**Author reply:** thank you for your suggestion and based on your comment we revised the the abbreviation and acronym accordingly.

**Introduction**

The introduction section provides an overview of the global prevalence of childhood obesity/overweight and highlights the increasing burden in low-income countries, specifically focusing on Ethiopia. Here are some comments on the introduction:

**Author reply:** Thank you for your input. We presented the statement of the problem starting from a global perspective, then focusing on Africa, and finally narrowing down to our specific study context. We aimed to describe the prevalence, risk factors, and burden of overweight in a hierarchical manner. Thank you!

Outdated references: The introduction section relies on references that are outdated, as indicated by the reference numbers (e.g., reference numbers 1, 2, 3, etc.). It is important to ensure that the references used are current and reflect the latest available data and research on the topic. Some of the reference also incorrectly cited. The authors should find the primary source and let the authors acknowledge them

**Author reply:** thank you for your suggestion and based on your comment we revised the reference by incorporating only the recent references thank you!

**Lack of global to local context**: The introduction does not provide a clear transition from the global context of childhood obesity/overweight to the specific local context of Ethiopia. It would be beneficial to link the global prevalence statistics to the situation in Ethiopia and highlight the unique factors contributing to childhood obesity/overweight in the country.

**Author reply:** thank you for your suggestion and based on your comment we revised the introduction section to include context from the global to the Ethiopian perspective, highlighting previous studies.

**Limited attention to childhood obesity in Ethiopia**: The introduction suggests that Ethiopia does not consider childhood obesity as an emergent public health concern and mentions limited attention given to this issue. However, they did not put the references for their investigation or justification, and it would be helpful to provide more context or evidence to support this claim and explain the potential reasons for the lack of attention.

**Author reply:** thank you for your suggestion and based on your comment we revised the introduction section to include childhood obesity as an emergent public health concern and mentions limited attention given to this issue with updated reference.

**Need for spatial analysis:** The introduction mentions that previous research in Ethiopia did not consider the spatial distribution of overweight/obesity and spatial regression analysis. It correctly highlights the importance of spatial analysis for policymakers to allocate resources effectively. However, it would be beneficial to provide more context on why spatial analysis is specifically relevant in the Ethiopian context and how it can contribute to addressing the issue.

**Author reply:** thank you for your fruit full comment and based on your comment we revised the introduction section by incorporating the need of spatial analysis for the study. Please have a look. Thank you!

**Research gap:** The introduction identifies a research gap by stating that few studies have explored community-level factors influencing overweight/obesity in Ethiopia. This highlights the need for the current study and its contribution to filling this gap. However, it would be helpful to provide more context on the specific aspects of community-level factors that have been underexplored in previous research.

**Author reply:** We appreciate your comment, which has guided the revision of our introduction. In response to identified gaps in existing literature, our study utilizes secondary data from the Demographic and Health Surveys (DHS), incorporating a comprehensive array of relevant variables. Our study emphasizes the underexplored community-level factors which included in the data were consider that influences on overweight and obesity in Ethiopia. By employing Geographically Weighted Regression (GWR), we analyze spatial variations in these factors across different regions, aiming to provide nuanced insights that inform targeted public health interventions tailored to local contexts.

**Importance of the study**: The introduction briefly mentions that the study aims to determine the spatial variations of overweight/obesity and its associated factors among under-five children in Ethiopia. It also emphasizes the potential implications of the study for policymakers, health planners, researchers, and health professionals. However, it would be beneficial to provide more specific details on how the findings of this study can inform policy and practice in Ethiopia.

**Author reply:** Thank you for your best and fruit full comment based on this we revised as, the study aims to investigate spatial variations of overweight/obesity and associated factors among under-five children in Ethiopia, utilizing Geographically Weighted Regression (GWR) with Demographic and Health Survey (DHS) data. The findings hold significant implications for policymakers, health planners, researchers, and health professionals. Policymakers can use the results to develop targeted policies and interventions to reduce childhood overweight/obesity rates across different regions. Health planners will benefit from insights to strategically allocate resources, focusing on areas with higher prevalence and specific risk factors identified by the study. Researchers can build upon these findings to explore causal relationships and refine intervention strategies, while health professionals can implement evidence-based approaches tailored to local contexts. Ultimately, these actions can collectively contribute to improving public health outcomes for under-five children in Ethiopia by addressing both prevention and management of overweight/obesity.

**Methods and materials**

**Study design, period, and area**

What is EMDHS

**Author reply**: thank you for your concern and for more understanding of the abbreviation we were incorporate in the abbreviation section as Ethiopian Mini Demographic Health Survey.

Please clearly define the study setting, and related concepts or feeding practices and sources in the country. It will help readers Try to put regionals states based on their order, and indicate there also another additional emerging region

**Author reply** Thank you for your suggestion and based on your comment we were describe the study setting clearly in the method section, thank you

**Source and study population**

The authors declared that their population was from 0-59 months. The question is

Body Mass Index (BMI) is generally not measured for newborns. BMI is a measure of body fat based on height and weight, typically used in older children, adolescents, and adults. For newborns, different measures are used to assess growth and development, such as weight-for-age, length-for-age, and weight-for-length percentiles. These measures help evaluate whether a newborn's growth is within the expected range for their age and sex.

BMI calculations require both height and weight measurements, which are not practical or accurate to obtain in newborn infants. Additionally, BMI is not considered an appropriate indicator of body fat for infants due to their rapidly changing body composition and growth patterns.

**Author reply:** Thank you for your concern and in our study the outcome was measured using the World Health Organization (WHO) standards for child growth monitoring. Specifically, they used the weight-for-height z-score, which is a reliable method for assessing growth and nutritional status in children. According to WHO guidelines, a child is classified as overweight or obese if their weight-for-height z-score exceeds +2.0 standard deviations (SD) from the mean. This approach allows for accurate monitoring of growth and development, ensuring that children who are at risk of overweight or obesity are correctly identified based on globally recognized standards. This method accounts for the rapidly changing body composition and growth patterns in infants and young children, making it a suitable alternative to BMI for this age group.

What about those children whose day of birth is missing or unknown.

How did you manage other missing values? The authors should put the detail regarding their management

**Author reply:** Thank you for your concern and we were handling missing data, imputation techniques are employed to estimate or replace missing values based on existing data. Methods like mean, mode, or median imputation replace missing values with summary statistics of observed data, assuming similarity among values. More advanced techniques include regression-based imputation, where missing values are predicted using relationships with other variables.

**Data collection tool and procedures**

In this section the author mentioned about other than the given topic

No sample size determination and sampling methods section

The authors need to create new session about sample size determination and sampling methods

Reference 14, 19…were not the appropriate references for the Data collection tool and procedures and outcome variable. Kindly use the correct one

**Author reply:** thank you for your suggestion and we revised it accordingly. Thank you!

**Data management and statistical analysis**

What does it mean “Using the STATA drop command in conjunction with a logical or conditional expression, we remove missing values from our analysis”.

**Author reply:** thank you very much for your input to improve the manuscript. Accordingly, we revised it clearly.

**Autocorrelation**

Why the authors applied “The outcome variable has a Bernoulli distribution”. Have you appreciated that the continuous nature was also used in your model? Why did not use the poison one

**Author reply:** thank you for your best concern. In our study, the decision to model the outcome variable with a Bernoulli distribution instead of a continuous distribution like Poisson was guided by the nature of the data and statistical considerations. The outcome variable in our analysis is binary, representing a dichotomous response (yes and no). A Bernoulli distribution is well-suited for such binary outcomes as it directly models the probability of a single binary event occurring. While the outcome variable is binary, our modeling approach incorporates continuous predictors or covariates to capture relationships with the probability of the binary outcome. This enables us to examine how these predictors influence the likelihood of the outcome, enhancing both the interpretability and predictive accuracy of our model. Comparisons during model selection supported the use of the Bernoulli distribution over alternatives like Poisson, aligning with the characteristics of our data and facilitating clearer interpretation of model results in the context of binary outcomes. Thank you!

**Results**

Do not use the new sentence with “of” and the like

Author reply: ok thank you!

How many unweighted participants did you included

**Author reply:** thank you for your concern and before weighting the participant’s number we have got 3,602. It was below the weighted participants.

Have the authors found primary clusters only in the SaTscan analysis

“Spatial scan analysis” what do you mean by “Spatial scan analysis” did you mean “Spatial SaTScan analysis”

**Author reply:** yes, we want to write as Spatial SaTScan analysis, so we were revised it clearly, thank you!

**Discussion**

The provided discussion contains valuable information about the geographical variations in overweight/obesity and related factors among Ethiopian children under the age of five. However, there are a few areas that could be improved:

**Consistency in referencing**: The discussion refers to various studies to compare the prevalence of overweight/obesity in Ethiopian children, but the references are not consistently cited using proper citation format. It would be helpful to ensure that the references are cited correctly and consistently throughout the discussion. There are also miss citations. The author should have the primary source for their citation

**Clarity in reporting findings**: While the discussion provides some numerical findings regarding the prevalence of overweight/obesity in different regions, it would be beneficial to present the findings in a more organized and structured manner. Consider using tables or concise bullet-point format to clearly present the prevalence rates and comparisons between regions and previous studies.

**Explanation of findings:** The discussion mentions various factors that may explain the observed variations in overweight/obesity, such as delivery via cesarean section, socioeconomic status, and urban residence. It would be helpful to provide more detailed explanations for each factor, including the underlying mechanisms and potential implications for interventions or preventive measures.

**Expansion of limitations and implications**: The discussion briefly mentions methodological differences, cultural feeding practices, and nutrition assessment methods as possible explanations for the observed discrepancies in overweight/obesity prevalence. It would be beneficial to expand on these limitations and discuss their implications for future research or interventions in more detail. Furthermore, it was not clear how and why authors excluded missing values

**Consistency in language and terminology:** Some sentences in the discussion use different terminology or language styles. It is important to maintain consistency in terminology and language throughout the discussion to ensure clarity and coherence

Write clearly the implication of your study findings to the policymakers

**Author reply:** thank you for your comment to improve the manuscript and we expand the discussion section, explanations of the findings, consistency of referencing and consistency of language and terminologies and we were not removed the missing value rather we handles it through imputation technique. Moreover we incorporate the implication of the study at the end of discussion section. Please have a look thank you!

**Reviewer comments 2**

**Abstract:**

1. Result : please support by statistics the following result “The spatial distribution of overweight/obesity among under-five children in Ethiopia was clustered”

**Author reply:** thank you for your essential comment and we revised accordingly.

2. Please support by statistics the following finding in the result “In the geographically weighted regression analysis, urban residence, cesarean section, rich households, and male children were statistically significant factors.

**Author reply:** Thank you for your concern and we accept your comment. The outcomes were derived using Geographic Weighted Regression (GWR) analysis, which identified significant factors respect to the regions. However, due to the abstract length limitations set by journal guidelines, these details could not be included.

3. conclusion: please put yours recommendation based on the result , means against the identified factors

**Author reply:** thank you and we revised as Targeted efforts led by the Ministry of Health and the Ethiopian Public Health Institute should prioritize regions in Ethiopia where factors like cesarean section delivery, higher wealth indices, urban residency, and gender disparities contribute significantly to childhood overweight and obesity. Policies must focus on implementing localized physical education programs and comprehensive health education campaigns through mass media to encourage active lifestyles and reduce sedentary behaviors among under-five children, with ongoing monitoring and community involvement to ensure sustained health improvements.

4. **what is the contribution of 12 authors in secondary analysis**

**Author reply:** Thank you for your concern and the 12 authors made significant contributions to understanding the spatial variation of obesity among under-five children in Ethiopia. They meticulously prepared and analyzed data from diverse sources, conducting descriptive and spatial analyses using Geographic Information Systems (GIS) and advanced statistical techniques like Ordinary Least Squares (OLS) and Geographic Weighted Regression (GWR). Their findings were visually represented through graphs and maps, highlighting geographical patterns and correlations between childhood obesity prevalence and factors such as urbanization and socio-economic status. Through their collaborative effort, the authors provided nuanced insights that are crucial for guiding targeted public health interventions and policy strategies aimed at reducing childhood obesity across different regions of Ethiopia.

**Background**

1. please put citation for the following statement “The majority of overweight and obese children live in developing countries, where the pace of increase is more than 30% greater than in industrialized countries.

**Author reply:** thank you for your critical comment and we revised by incorporated the reference.

2. The following statement is not clear please rewrite “Globally, the prevalence of children under five who are obese was 7% in 2012, and by 2025, it was expected to drop to less than 11% (5)” can we say 7% to 11 % is drop???.

**Author reply:** thank you for your essential comment to improve the manuscript and we revised as Globally, the prevalence of obesity among children under five was 7% in 2012, and it was projected to rise to nearly 11% by 2025.

3. Please rephrase the following statements “From 5.4 million in 1990 to 10.3 million in 2014, the number of overweight and obese children in Africa has almost doubled (2, 6)”

**Author reply:** thank you for your essential comment to improve the manuscript and we revised as In Africa, the number of overweight and obese children has almost doubled, increasing from 5.4 million in 1990 to 10.3 million in 2014.

4. Rephrase the following statement as “6.8% of children between the ages of 0 and 59 months are overweight or obese, according to 26 Demographic and Health Surveys that have been carried out in the SSA since 2010 (7) “.you can rephrase as : according to the Demographic and Health Surveys conducted in SSA, about 6.8% of children between the ages of 0 and 59 months were overweight or obese in 2010.

**Author reply:** Thank you for your comment and suggestion. As a result we were revised the above sentence clearly as according to the Demographic and Health Surveys conducted in SSA, about 6.8% of children between the ages of 0 and 59 months were overweight or obese in 2010.

**Generally, the introduction is not well written, incoherence, and many of the statement were written without citation, my recommendation for the authors, please remove unnecessary details and put your evidence or citation for some of statements that reported without citation.**

**Author reply:** thank you for your comment to improve the manuscript before publication, as a result we revised the introduction section properly. Please have a look.

**Methods:**

1. **When do you score "1," else marked as "0" for the outcome variable “Overweight/obesity “**

**Author reply:** Thank you for your concern and during the data preparation and cleaning stage of our analysis, we meticulously categorized the outcome variable to ensure clarity and accuracy in our findings. This process involved structuring and organizing the outcome variable into distinct categories or classes that reflect the different possible outcomes or levels being studied.

**The following statement is not clear “STATA was used to tabulate the weighted proportions of outcome variables and prospective predictor variables, which were then exported to Excel and imported into ArcGIS 10.6 for additional analysis”, please remove**

2. How the authors handle the two levels hierarchy nature of MEDHS data (multi-stage stratified cluster). That the Level one units were individual children in households and level two units were enumeration areas (community level). That Level one children were nested in the households, then the households were nested at the next higher level of enumeration areas ( community level). With is reality, Is Geographic Weighted Regression model is the appropriate model to identify the individual level and community-level factors as you claim in the bottom of your introduction???. I firmly recommend the authors to run multilevel mixed effect logistic regression rather Geographic Weighted Regression model to identify the individual level and community-level factors as you claim to identify factors at respective level.

**Author reply:** In analyzing the MEDHS data's hierarchical nature where children are nested within households and households within enumeration areas the choice between Geographic Weighted Regression (GWR) and traditional multilevel mixed-effects logistic regression is pivotal. While multilevel models are conventional for handling nested data structures, GWR offers distinct advantages in capturing spatially varying relationships between predictors and outcomes. This approach is particularly beneficial for MEDHS data, where community-level factors may vary significantly across geographic areas, influencing health outcomes in diverse ways. By employing GWR, researchers can uncover localized effects that traditional models might overlook, thereby enhancing the precision of identifying individual-level and community-level factors affecting health.

Advocating for GWR in this context acknowledges its capacity to accommodate spatial heterogeneity and account for regional variations in health determinants. Unlike multilevel models that assume consistent effects across all locations, GWR allows for the exploration of how predictors influence health outcomes differently across different regions. This spatially explicit approach not only improves the accuracy of findings but also supports the development of targeted public health strategies tailored to specific geographic areas. Thus, while multilevel mixed-effects logistic regression remains valuable, the adoption of GWR in analyzing MEDHS data underscores its potential to reveal nuanced insights into health disparities and inform more effective intervention efforts at both individual and community levels.

3. How you weight the variables

**Author reply:** To appropriately weight variables in the analysis of MEDHS data, it is crucial to generate a weight variable (wt) that reflects the inverse of each observation's sampling probability, often calculated as wt = v005 / 1000000, where v005 represents the sampling weight provided in the dataset. This weight adjustment compensates for unequal probabilities of selection and non-response in the survey design, ensuring that statistical analyses accurately represent the broader population. When analyzing variables such as the prevalence of childhood overweight (overweight), applying the weight (iw=wt) in commands like tabulate v001 overweight [iw=wt] adjusts the results to reflect population-level estimates, thereby enhancing the reliability and validity of findings for informing health policies and interventions based on MEDHS data.

**Results**

4. Please, specify the individual level and community-level factors **separately in result**

**Author reply:** Thank you for your suggestion to improve the manuscript before publication, however if we put them separately it seems like multilevel analysis and in our point of view it is better to present as in the manuscript described.

**Discussion**

5. generally the discussion need rewriting and grammar correction

**Author reply:** Thank you for your critical comment and we revised the discussion section clearly accordingly. Please have a look.

6. Please write the clinical and public health importance of this study ,just at end of discussion

**Author reply**: Thank you for your critical comment and we incorporate the implication of the study for clinical, public health and policy makers in the discussion section clearly. Please have a look. Thank you!

***Thank you!***

***Correspondence author: Agmasie Damtew Walle***

***PLOS ONE academic editor***
