# Supplementary material for: Spatial variations and predictors of overweight/obesity among under-five children in Ethiopia: A geographically weighted regression analysis of the 2019 Ethiopian Mini Demographic and Health Survey
Source: PLoS One. 2024 Oct 14;19(10):e0312025. doi: 10.1371/journal.pone.0312025 (PMC11472954; doi:10.1371/journal.pone.0312025)
Supplement: Attachment — Submitted filename: Editor Comments- PONE-D-23-34960 Article.docx [file pone.0312025.s005.docx]

**PONE-D-23-34960R1**
**Spatial variations and associated factors of overweight/obesity among under-five children in Ethiopia: a geographically weighted regression analysis of 2019 DHS data**

**Article**

**Editor Comments:**

The manuscript presents an interesting study on the effects of inulin on Spatial variations and associated factors of overweight/obesity among under-five children in Ethiopia: a geographically weighted regression analysis of 2019 DHS data. The manuscript is well written and comprehensive, and the methodology used seems robust as the sample is large and covers many locations in Ethiopia. However, there are some points that require revision to enhance the clarity and scientific accuracy of the manuscript.

- The introduction section provides a good overview of the global and regional context of childhood obesity. However, I suggest adding more recent data or references to give a more current picture of the situation. I would also recommend moving from the global context to the specific local context of Ethiopia to understand the factors contributing to obesity in Ethiopian children.
- In the results section, clarify the reporting of statistical analyses. For example, when discussing clustering patterns (Moran’s I and Z scores), consider providing more context on what these values indicate in practical terms.
- The section on “Factors influencing spatial variation in obesity/overweight” could benefit from more clearly separating individual-level and community-level factors, or at least providing a clearer explanation of why these factors are presented together.
- In the discussion section: I suggest providing a more explanation of the possible mechanisms underlying the associations of factors such as cesarean delivery, urban residence, and wealth index. These factors may provide a better explanation of childhood overweight/obesity.
- Finally, I suggest that rather than summarizing the main findings of the research in a general way in the conclusion, highlight how the study’s insights can be used to develop public health strategies to combat childhood obesity in Ethiopia, and provide more specific recommendations that policymakers can take based on the study’s findings.
- Ensure that all abbreviations are defined on first use and check for consistency throughout the manuscript.

Following the suggested comments, the manuscript will become more clear and impactful about the spatial patterns of childhood obesity in Ethiopia.

**Decision Recommendation:**

I recommend accepting the manuscript after minor revision.

Best regards,
