# Supplementary material for: Spatial variations and predictors of overweight/obesity among under-five children in Ethiopia: A geographically weighted regression analysis of the 2019 Ethiopian Mini Demographic and Health Survey
Source: PLoS One. 2024 Oct 14;19(10):e0312025. doi: 10.1371/journal.pone.0312025 (PMC11472954; doi:10.1371/journal.pone.0312025)
Supplement: Attachment — Submitted filename: point by point response.docx [file pone.0312025.s006.docx]

**Date: 10/09/2024**

## **Dear editorial board member (s) of** **PLOS ONE journal**

The authors have been recalled to revise the manuscript entitled “Spatial variations and associated factors of overweight/obesity among under-five children in Ethiopia: a geographically weighted regression analysis of 2019 DHS data” identified by unique manuscript number: **PONE-D-23-34960,** which was submitted for publication. So, we received the **editor(s)’ and reviewer's minor revision** comments for the improvement of the manuscript before its publication.

Thank you for receiving comments, suggestions, directions, and questions from the editor and reviewer. As we said usually, we are very happy in receiving constructive and valuable comments for making a better manuscript. Accordingly, we have considered all the comments, questions, directions, and suggestions and provided a point-by-point response letter.

Finally, we have submitted all the required documents in their revised form. We hope that we have addressed all the suggestions, directions, and raised questions and if you believe that point(s) is not addressed, please let us know.

**Thank you very much to all editor (s), and reviewers**

**On the behalf of the authors**

**Yours sincerely,**

**Correspondence author**

**PLOS ONE academic editor**

**Point-by-point response letter**

**Editor comments**

The manuscript presents an interesting study on Spatial variations and associated factors of overweight/obesity among under-five children in Ethiopia: a geographically weighted regression analysis of 2019 DHS data. The manuscript is well written and comprehensive, and the methodology used seems robust as the sample is large and covers many locations in Ethiopia. However, there are some points that require revision to enhance the clarity and scientific accuracy of the manuscript.

- The introduction section provides a good overview of the global and regional context of childhood obesity. However, I suggest adding more recent data or references to give a more current picture of the situation. I would also recommend moving from the global context to the specific local context of Ethiopia to understand the factors contributing to obesity in Ethiopian children.

**Author reply:** Thank you for your fruitful comments and suggestion and based on your comment we revised the introduction section by incorporating global to Africa to Ethiopian context clearly please have a look.

- In the results section, clarify the reporting of statistical analyses. For example, when discussing clustering patterns (Moran’s I and Z scores), consider providing more context on what these values indicate in practical terms.

**Author reply:** Thank you for your essential suggestion and we describe the indication of the Morans I and Z score in the result section line 346-349 as well as in the method section of spatial analysis heading, line 270-272, please have a look the highlighted colour in the ***tracked change of the manuscript***.

- The section on “Factors influencing spatial variation in obesity/overweight” could benefit from more clearly separating individual-level and community-level factors, or at least providing a clearer explanation of why these factors are presented together.

**Author reply:** Thank you for your concern and valuable feedback on the section Thank you for your feedback on the section “Factors Influencing Spatial Variation in Obesity/Overweight.” In this analysis, which utilizes Geographically Weighted Regression (GWR), we focused on identifying spatial variations in how different factors influence obesity/overweight rather than performing a traditional multilevel analysis. GWR allows us to examine how the impact of these factors varies across different geographic areas, providing insights into localized patterns and interactions. This approach is well-suited for capturing the spatial heterogeneity of factors and understanding how their effects differ regionally. To clarify, we were include a detailed explanation in the revised manuscript of how this method specifically addresses variations across different areas in introduction section clearly. Thank you!

- In the discussion section: I suggest providing a more explanation of the possible mechanisms underlying the associations of factors such as cesarean delivery, urban residence, and wealth index. These factors may provide a better explanation of childhood overweight/obesity.

**Author reply:** Thank you very much for your critical comment to improve the manuscript before publication and we revised the given comments in discussion section accordingly. Please have a look the discussion section of the manuscript.

- Finally, I suggest that rather than summarizing the main findings of the research in a general way in the conclusion, highlight how the study’s insights can be used to develop public health strategies to combat childhood obesity in Ethiopia, and provide more specific recommendations that policymakers can take based on the study’s findings.

**Author reply:** Thank you very much for your suggestion to improve the manuscript before publication and we revised the conclusion section accordingly. Please have a look the conclusion section of the manuscript.

- Ensure that all abbreviations are defined on first use and check for consistency throughout the manuscript.

**Author reply:** Thank you for your valuable suggestion regarding the definition and consistency of abbreviations. We have reviewed the manuscript to ensure that all abbreviations are defined upon first use and have checked for consistency throughout. As a result, all of them were consistent and defined clearly.

**Reviewer 3 comments**

**Reviewer #3:** Title: Please write DHS's full name in the title; it is recommended that abbreviations be avoided.

**Author reply:** Thank you for your fruitful comments and suggestion and based on your comment we revised as “*Spatial Variations and Predictors of Overweight/Obesity among Under-Five Children in Ethiopia: A Geographically Weighted Regression Analysis of the 2019 Ethiopian Mini Demographic and Health Survey* “
**Introduction:** Kindly add the clear SMART objectives of the study at the end of the introduction/ background.

**Author reply:** Thank you for your fruitful comments and suggestion and based on your comment we revised the introduction section by incorporating SMART object at the end of the introduction section. Please have a look.

**Methods:** The study omitted several potentially influential factors, such as dietary habits, breastfeeding practices, maternal nutritional status, and physical activity levels. Including these variables would provide a more comprehensive analysis of the determinants of childhood obesity.

**Author reply:** Thank you for your valuable comments to improve the manuscript before publication. Based on your feedback, we have revised the conclusion section by suggesting that future researchers include the factors you mentioned to enhance the representativeness of the findings. Kindly review the updated conclusion section.

The paper could benefit from more detailed descriptions of the statistical methods, particularly the GWR analysis. Readers unfamiliar with these techniques might find that the current explanation needs to be revised. Including a brief introduction to how these methods work and why they were chosen would enhance understanding.

**Author reply:** Thank you for your valuable feedback. Based on your suggestion, we have revised both the **Introduction** and **Methods** sections to provide a more detailed explanation of the Geographically Weighted Regression (GWR) analysis. In the introduction, we now include a brief overview of how GWR works and the rationale for selecting this method. In the methods section, we have expanded the description of the statistical process, explaining the steps taken in the GWR analysis, including how the model accounts for spatial heterogeneity and the reasoning behind the chosen parameters. These additions aim to enhance understanding for readers unfamiliar with GWR techniques.

**Results and Analysis:** While the study identifies significant factors like urban residence and cesarean delivery, further adjustments for potential confounders (e.g., parental BMI, socio-economic status) could refine these associations

**Author reply:** Thank you for your valuable comments to improve the manuscript before publication. Based on your feedback, we have revised the conclusion section by suggesting that future researchers include the factors you mentioned to enhance the representativeness of the findings. Kindly review the updated conclusion section.

**Conclusion:** The conclusions could benefit from including more actionable steps or strategies for stakeholders. Suggestions for immediate actions based on the findings would make the study more practical and applicable.
Highlighting specific areas for future research, such as longitudinal studies to track changes over time or interventions tailored to specific regions, would provide a clear direction for advancing the understanding of childhood obesity.

**Author reply:** Thank you very much for your suggestion to improve the manuscript before publication. We have revised the conclusion section to incorporate your feedback and have made the necessary adjustments for clarity. Please review the updated conclusion section of the manuscript.

With regards!

Agmasie Damtew Walle

Corresponding author
